# *DeusEx* Saved My Life: A Feminist-Autoethnography of Video-Gaming Through Major Depressive Disorder

**DOI:** 10.1177/10497323241307193

**Published:** 2024-12-17

**Authors:** Nicholas Norman Adams

**Affiliations:** 1 The School of Health, 1018 Robert Gordon University, Aberdeen, Scotland

**Keywords:** major depressive disorder (MDD), gaming therapy, video games and depression, autoethnography, feminism

## Abstract

Autoethnographic accounts of mental illness (MI) are sparse in academic scholarship, despite generating valuable insights into how MI can be experienced and coped with in real-life contexts. First-person accounts from men are especially lacking, possibly linked to historic trend for masculine stoicism stifling male MI discussions. Some scholarships explore video-gaming as a positive, escapist aid benefiting individuals experiencing major depressive disorder (MDD). However, no research exists presenting in-depth perspectives on possible positive effects, self-identified and articulated by actors engaging with gaming to cope with MDD. This research adopts a novel qualitative perspective, representing an in-depth autoethnographic examination of my experiences playing the personal computer game *DeusEx*, during a period of my life where I was under treatment for MDD. My positions as both a psychologist specializing in research prioritizing feminist theory as applied to understand men, masculinities, and mental health, and someone themselves recurrently treated for MDD over longer than 22 years, construct a unique dual-positionality perspective. Explicit discussions of my MDD experiences and my experiences concerning the value of video-gaming as a positive, escapist aid during MDD are presented, alongside personal deconstructions of the lasting influences of *hegemonic masculinity* upon men speaking up about MI. Arguments are presented for future scholars utilizing autoethnographic methods to generate realist perspectives, normalizing mental health discussions, particularly the sharing of underrepresented male experiences. Implications for future scholarship, building upon learnings generated by this research, are developed and put forward.

## Introduction

### Background to Writing

When conceptualizing this research, what struck me most was the absence of academic autoethnographic accounts of mental illness (MI)^1^ published. While research examining depression, anxiety, and other illness is plenty, reflexive mental health (MH) perspectives—particularly from men—are lacking. Male perspectives may be sparse for several reasons, including recognition that men may feel less likely to openly discuss experiences for fear of being perceived as “weak,” “fragile,” or “lesser men” (Galasiński, 2008; Wagner & Reifegerste, 2024). Male scholars who have written candidly about their depressive experiences recognize the difficulties in doing so, and how negative self-perceptions of MDD as “exaggerated” or “self-inflicted” can be intensified by writings:

Writing about one’s own depression is difficult. Depression is partly constituted by a sneering “inner” voice which accuses you of self-indulgence – you aren’t depressed, you’re just feeling sorry for yourself, pull yourself together – and this voice is liable to be triggered by going public about the condition. (Fisher, 2014a, p. 1)

For many years, I have been inspired by the writings of the late Mark Fisher, a cultural theorist whose 2014 book—published only 3 years before he took his own life—was titled: *Ghosts of my Life* (Fisher, 2014b). Not since reading John Healey’s 1988 masterpiece autobiography *The Grass Arena* (Healy, 2008) had I felt that an account of struggle and despair had been so carefully, honestly, and rigorously analytically constructed. This utilizing an autoethnographic lens revealing real-life lived experiences with such impactful luminosity, so as to generate unparalleled relation, understanding, and self-reflection. *Ghosts of my Life* inspired me to construct this paper; Fisher’s work delves into topics exploring influences and juxtapositions between contemporary society and MH (Fisher, 1999, 2014b, 2018). I have tried to develop my accounts of MDD factoring a brutally honest and open perspective, with the goal of operating in some small way toward attempting to normalize men sharing their perspectives and experiences of poor MH. This is in the hope that together a growth in autoethnographic perspectives can be produced, from which learnings, interventions, and observations may generate positive support opportunities, and theoretical challenge and progress.

### Video Games, Mental Health, and Escapism

Some research explores video games as having positive benefits for those experiencing MDD—however, positive affect is suggested as tentative (Granic et al., 2014; Russoniello et al., 2009, 2013, 2019). Little scholarship explores the concept of escapism from symptoms as a primary facilitator of affect (Hagström & Kaldo, 2014). Conversely, some studies have suggested “overescapism,” disconnecting from reality, as linked to video-gaming, suggesting negative MH influence (Kaczmarek & Drążkowski, 2014). Escapism is a difficult concept to measure, requiring understandings of social actors’ temporal-and-fluctuating state(s) of mind, feelings, perceptions, mood, and emotions, and how subjective cognition, perception, and well-being are perceived as altered (by the actor) following engagements with different stimuli (Stenseng et al., 2021). Additionally, those studied must also possess enough self-awareness and objectivity to ascertain (and articulate) whether alterations in experience and mood are positive or negative, following exposure to video-gaming (Kardefelt-Winther, 2014; Kırcaburun & Griffiths, 2019; Warmelink et al., 2009). These considerations render the examination of experiences of gaming-mediated escapism from anything other than a first-person-perspective challenging. By default, games are designed around a process of interaction and interface of human–machine signals. Therefore, the game player is centered in the activities of gaming as a natural first-person interaction and is best situated to describe perceived effects from this intended perspective (Chua et al., 2013; Klimmt et al., 2009, 2010).

Linked to the above considerations, autoethnography (AE) is underrepresented within social sciences, especially studies of MH, yet represents a powerful and precise method for centering lived experiences as a primary mode of phenomenological examination (Foster et al., 2006; Liggins et al., 2013; Wakeman, 2021). Despite this potential, within studies exploring the role of video-gaming upon MH, autoethnographic perspectives are largely absent. It is rare for an ethnographer—who is also a psychologist—to self-examine their own MH experiences to generate scholarly insights on a topic of personal importance. However, in matters of MH, where describing positive escapism from symptoms, and mechanisms by which this may be achieved is the primary research focus; AE represents an unsurpassed method in appropriateness, allowing for analytical, reflective, and reflexive cataloguing and examining of lived experiences (Smith, 2015; Tolich, 2010).

The focus of this paper is to develop and comprise a detailed AE of my own experiences of gaming: playing *DeusEx*, while experiencing medically diagnosed major depressive disorder (MDD). I utilize feminist-AE to examine my experiences of video game–induced escapism as a positive tool, an immediate, readily accessible means to “cope” with primary symptoms of MDD. Additionally, this research allows myself, in the dual researcher–researched role, to analytically explore my MH journey via same lens, generating retrospective insights into conceptualizations of illness, help-seeking, and resistance linked to the lasting influence of *hegemonic masculinity* propagated both in some video games and wider society, generating salient observations.

The following sections present the methodology for this study. Then this study follows a detailed exploration of my own MDD experiences, dealing with MDD “recovery,”^2^ and the role of *DeusEx* and escapism within this process. A discussion follows that reinterprets my personal experience through an analytical feminist lens, developing novel sense-making for the role of video games as a supportive, escapist aid and deconstructing the influence of *hegemonic masculinity* upon men’s MH.

## Methodology: Feminist-Autoethnography

Autoethnography refers to conducting and analyzing own experiences; adopting a self-reflective and reflexive “inner lens” that has been employed as a central methodology within feminist scholarships to explore and elicit a range of emotive, societal, and structural perspectives surrounding intersections of inequality and experience (Crawley, 2012; Ettoree, 2016). Some define AE itself as a fundamental feminist practice (Allen & Piercy, 2005; Crawley, 2012; Ettorre, 2016; Taylor & Coia, 2020).

Contemporary feminism focuses on recognizing inequalities (Braithwaite, 2002; Connell, 2024; Lotz, 2007; Tarrant, 2009). Much thinking has applied evolving post-structuralist, feminist theory to examine masculinities, diverse intersectional constructs of being, representing identities of male-identifying social actors (Anderson, 2023; Connell, 2020; Connell & Messerschmidt, 2005; Gardiner, 2004; Kimmel, Hearn, Connell, 2004; Sabo, 2005). Resultantly, theory has developed as transdisciplinary feminist frameworks examining links between men’s conceptualizations and constructions of masculine identities and men’s behaviors, actions, and intentions (Adams, 2022, 2023b; Bridges & Pascoe, 2014; Connell, 2020; Darroch et al., 2023; Haywood & Mac an Ghaill, 2003).

Some scholars have successfully utilized adapted autoethnographic approaches—including feminist-AE—as methods for self-examining masculinities, gendered behaviors, and reconfigurations in men’s behaviors and practices. For example, Henry (2017) in *Hit Him Again* presents a harrowing autoethnographic account of “learning violence” (p. 96), recounting stoicism and repression propagated and normalized during youthful inductions to childhood violence. Henry channels a personal AE account to explore didactical linkages between masculine notions, violence, and rejections, arguing, following an immediate and graphic recounting of exposure to familial violence: “[these] performance[s] of gender empowers men to assert their self-importance as if in direct competition with those around them” (p. 96).

Similarly, Strasser (2016) invokes AE to explore restrictive familial bonds; emotional stoicism and suppression constructed by contextually normative masculine “dominant models” of “fathering” that harm intimacy (p. 1). Strasser explores changing masculine norms, communicative acceptance, and *new closenesses* that alter and reconfigure previously dominant masculine fatherly narratives, shifting sense-making toward new identity notions surrounding his relationship and interactions with his father. This following the shared experience of the death of a mutual friend.

Drummond’s (2010) masterful AE exploration of the construction of masculine narratives, identity, and actions involving sport and the physical body, as an elite triathlete, presents powerful examples of the interplay between societal expectations and the normalizations of specific masculine identities and physicalities networked with sport activity. This alongside mutual and symbiotic constructions of identities and physicalities as reproductions and challenges of masculinities as abilities, as the body evolves and ages. Drummond explores the heavily “hypermasculinised” (p. 7) influence of sport, connecting this to triathlon events defined as “man’s event[s]” due to the socially perceived physical requirements; endurance and discipline advertised as necessary to complete these (p. 7). This thinking is linked to examination of “bodywork” involved to maintain competitive male weight for such events, highlighting bodywork as reflective of fulfilling identity as an elite athlete but also carrying paradoxically detrimental bodily effects: these processes paradoxically replicating *and challenging* masculine notions of strength, stability, and bodily prowess:

My body is constantly in pain. My joints continually ache. […] I have lost every toenail, and they regularly bleed.

[…]

… while being a successful triathlete was important to me in terms of my masculine and self-identity, the sport itself had become a conduit for change in terms of my physical and emotional body. (p. 7)

As such, and as observed in other scholarship (Adams, 2023a), Drummond’s AE journey of reproduction for some masculine identities serve to paradoxically and simultaneously place under threat the very means by which such masculinities are produced and defined: the physical body.

My own feminist, AE investigatory lens links with the above works, acknowledging post-structural feminist *men and masculinities* scholarship as linked to this research and examining the gendered nature of MH, MDD, and video-gaming. Contemporary feminist theory recognizes nexuses of privileges occurring for different intersectional positions and societal spheres, while also recognizing oppression and inequalities. Arguably one of the only societal arenas within which men appear to be structurally disadvantaged is within the arena of openly discussing, seeking, and acquiring MH supports. Indeed, scholarships have identified pervasive stigmas attached to men “speaking-up” about MH struggles, occurring for numerous reasons dependent on context and social environment (Kua, 2009). Despite efforts to increase men’s discussions and help-seeking, trends for suppressing open dialogues, early and immediate supports continue to demonstrate stigma, reluctance, and masking (Butler, 2023; Miller et al., 2023; Sagar-Ouriaghli et al., 2023; Shepherd et al., 2023). An encompassing canon of recent scholarship links stigma with notions of *hegemonic masculinity*, considering help-seeking as weak and men perceiving themselves undeserving of help, or feeling requirements to attempt to “fix” their MH themselves (Galasiński, 2008; Keum & Ogrodniczuk, 2023; Reily et al., 2023; Silvestrini & Chen, 2023; Wagner & Reifegerste, 2024).

Structural stigma is itself a product of patriarchal processes. The role of *hegemonic masculinity* maintains a historical status quo of emotional stoicism *haunting* contemporary sense-making, as a shadow of past societal imprinting and norm-reproductions. To deconstruct this, *hegemonic masculinity* (HM) represents theory developed by R.W. Connell, giving language to discuss the normative acceptance of specific masculine ideology: emotional stoicism, strength, “toughness,” resilience, and competitiveness as archetypal male constructs (Connell, 2020). Connell is careful to suggest criteria for determining HM: performances of these behaviors—to be classified as HM—must operate to subordinate women and/or marginalized men via modes of attaching male identities to notions of femininities as a negative construct, thus propagating a damaging, normative patriarchal hierarchy with HM-congruent behaviors positioned as the ideal. This is a factor that has been overlooked by some past scholars utilizing HM theory, resulting in *slippages* (Lucy, 2024). HM manifests as an ideological construct; Connell describes this as a hierarchal configuration of masculine social tiers: the hegemonic, the complicit, and the subordinated. Connell argues that while HM represents the most revered and “accepted” construct of Western masculinity, it is not necessarily the most-subscribed. However, it is the construct most men position themselves in relation to. Men who subscribe to HM embody and reproduce key constructs of the identity as behaviors, attempting to position themselves as archetypal “men.” Men in complicit support of hegemony perform some behaviors, or perform behaviors neutral to subscription, but such behaviors lend complicit support to HM and its normalization, as these are not counter-hegemonic or do not pose challenges against HM. Finally, some men represent a tier of subordinated masculinities, these subscribing to notions of masculine identity that are converse to—allegedly misaligned—with notions or ideologies congruent with HM and its complicit support. Thus, these men are positioned as “less masculine,” operating in opposition to the dominant, most recognizable, and revered archetype of western male identity. This is despite these masculinities representing equally valid and legitimate constructs of intersectional being (Connell, 2020).

A dual-feminist-lens informed by AE and HM is utilized in this work, primarily to spotlight the first-hand masculine perspective: reveal inequalities and power dynamics with regard to linkages between gender and MH, that is, MH and MDD as uniquely experienced by men. Importantly, societal influences and impacts of HM as upheld and imparted—via social conditioning—and normative patriarchal structuring of society are acknowledged and explored within deconstructions of my own experiences, amplifying the feminist *HM* perspective.

### Validity, Triangulation, and “Researching the Self”

It is important to be clear about the process of conducting “self-research.” Although many autoethnographies are constructed, at least in part, from the memories of researchers, maintaining validity in AE is equally important as with other methodologies that do not explicitly focus on researching the self. The recollections of my own experience in this work are informed by several triangulated sources. Firstly, my memories of my experiences which, as with all memories, could be argued are naturally open to confabulations and misremembering. However, I would argue that experiences of MDD for many and especially concerning MDD first-experience and first-diagnosis have a certain *flashbulb* quality (Budson & Gold, 2009; Conway, 2013), memories that remain, clear, ingrained, easily recoverable, and less open to confabulations and processes of “memory-fade” compared with others. Indeed, it could be equally argued that the traumatic nature of MDD first-experience/diagnosis often qualifies as PTSD, similarly lending to the memories of this time as imbued with an effervescent “rapid-recall” quality (see Olszewski & Varrasse, 2005).

Secondly, my narrative draws on my personal notebooks from this time, which contain writings, but mostly drawings, poems, music-writings-and-lyrics, shorthand notations-to-myself, stuck-in artifacts, pictures, and cut-outs. Reviewing these has been challenging but has ultimately enhanced memory-recall and led to the validation of some memories recorded in detail in this work, linking back to sense-making on emotions occurring within the temporality explored. Some of these writings have been matched against various digital notations and authorship I have kept (and still engage with and develop) since I was a teenager, allowing for a gradual building of the “rich depictions” of experience described in this manuscript.

Thirdly and finally, one of the key sources of material aiding construction of this work has been “field recordings.” Since my early teens, I have been fascinated by the medium of audio-recording and—at age 40—have now amassed a large collection of analogue and digital audio-recordings of music, thoughts and memories, audio-notations, and reflections. Some of these have been lost to time, but much has been digitized and remains. Interestingly, my notations regarding my experiences of this time are preserved and exist in analogue format on FE90 cassette (Dictaphone and four-track) tapes, and I was able to listen back to these during the process of developing this work, using some legacy audio equipment.

The above combined materials are essential for strengthening validity of the autoethnographic tapestry, allowing for comparative triangulation-led analysis between memories and different validation sources, developing new—retrospective—reflective insights, and providing manifold perspectives to facets of lived experiences that bridge my current analytical self to historic occurrences. However, and with transparency, it is important to remember these materials also have a “real world” function and application. I reviewed the above data sources initially prior (and then during) the authoring of this manuscript for a different reason—upon realization my novel present medication plan, which I have written about previously (Adams, 2024), was waning in effectiveness and I was experiencing MDD-onset once again. These materials provide a patient-led reflective data source—more detailed and personal than any clinical record—that I can revisit, add to, and utilize to identify effects on mood, coping, and sense-making regarding illness, medications, and forward, lateral, or backward progress-making in my ongoing journey.

Concurrent to the above theme of validity, some suggest requirement for scholars utilizing AE to include within their work “hard” references and explicit “snapshot” components of diary entries and similar materials (Choi, 2016). This poses, firstly, an interesting quandary regarding making sensitive (and personal) materials “forever public” and indeed raises ethical considerations for how the publicity of such personal materials may later carry effects over the researcher. Some considerations similar to this have been explored in existing scholarship (Haynes, 2011), but I argue direct exploration of this *forever public* position is necessary to acknowledge the digital realities of late-modern society. A second quandary arises when considering “how valid” such materials may be in tangibly “validating” the researcher’s AE experience when viewed—without context—by others. For example, within one of my diaries, amidst nearly a month of blank pages is stuck a blurred instant-print photograph of an electricity pylon malfunctioning, showing a bluish set of sparks. This pylon has significant meaning to me, as I was able to see it from the window of my parents’ spare bedroom, where I spent much time in bed—stagnant—when visiting them. To me, the photograph represents a deep frustration and inertia, embodying one of the only dynamic aspects of stimuli I saw during this time: the pylon sparking and humming, slowly breaking down without anyone noticing or intervening, yet appearing, on the surface to still fulfill its core function. Thus, there is a deep metaphorical connection to my own feelings and experiences at the time, and this was my reasoning for taking the photograph, journaling this, and circling around it in black felt pen, without any added words—yet, this meaning; *this memory* is invisible to others. As such, for the purposes of this research and factoring the aforementioned ethical dilemma on “forever sharing” personal materials, I have decided against sharing direct extracts from my archival diaries and notations, recognizing that these are largely symbolic in nature and to explain each artifact would be prohibitive within the word count of this manuscript. Instead, I have wound the narrative of the following sections around a set of entries depicting the triangulated mixed-media that I used at the time to record my sense-making of MDD, ensuring validity and reliability, but balancing this with required protections for myself and readers.

## *DeusEx* Saved My Life

It is a daunting undertaking to write candidly about one’s own experiences of depression. In addition to considerations raised by Fisher (2014a), that depressive experiences are somehow one’s own fault or resultant of a lack of ability to “pull [oneself] together” (p. 1), there is the additional public concern that shared experiences will be “out there” forever, always linked to the author as a public point of reference. However, in weighing up the possible wider benefits versus possible personal “cons” or stigmas generated by writing this, any de-stigmatization and normalization occurring from this work calling for open discussions and increased uptake in men using AE to share their experiences firmly outweighs any negatives facing myself.

It’s difficult to put into words how “challenging” my own experiences of MDD (and linked diagnoses) have been over the last 22 years and how they have affected my life. I rarely speak about these experiences with anyone. While I was first diagnosed with (severe) MDD at 18 years old (in 2002), my experiences with depression and MI long predate this and are linked to other diagnosis^3^ and past circumstances. However, as this AE primarily concerns my first spell of MDD, which began when I was 18 years old, “ending” (or more accurately going into temporary remission) when I was 22, it is perhaps fair to describe this as the most memorable, devastating, and frightening experience of MDD I have had to date. This is despite being recurrently treated for MDD (and later treatment-resistant MDD) a multitude of times since then and negotiating complex additional diagnoses following. This is down to several factors but predominantly a fear of the unknown. In my now-significant experience, the “normal” presentation of MDD for me links to very low mood, anxiety, obsession over specific fields of interest, low-self-worth, and almost no ability to sleep but coupled, I suppose, in some form of balance, with a remaining of energy; engagement in activities such as exercising, writing, and working, which strangely appear to have their motivations and impetus for engagement spared, despite the pleasure when engaging in these activities reducing to zero.

### My Experiences With MDD

When I experienced my first spell of MDD, back in 2002, I gradually, over a period of around 6 months leading up to my formal diagnosis, near-completely stopped engaging with anything in my life. I was then (and still am) a swimmer, swimming multiple times a week since a very young age. Therefore, it seems fitting I utilize swimming metaphors as a first port of call for describing the sensations of depression. When depression first set in, my swimming completely stopped; each training session increasingly hampered by the sensation that I was pulling more than my body in the water, that I was pulling an additional body behind me. So, I gave-up swimming. When engaging in other activities, this “dragging” sensation gradually manifested into permanence. I felt like I was carrying the same weight of myself on my back always. I felt a constant, crushing notion of being completely weighted down, of being suffocated by a dark figure of immeasurable pressure that could crush the air from my lungs, pressure my heart into a rapid-pounding anxiety, and squeeze my muscles and bones into an inertia: someone sitting on your shoulders, with their legs wrapped firmly around your chest—a feeling impossible to shake off. It felt like this 24 hours a day: constant. It was a sensation like swimming underwater following a dive, of hitting the diving pool, breaking the surface of the water and feeling the pressure mount as the body flows downward from the surface, the pressure crushing the body and the cold and darkness of the water intensifying, muffling the rest of the world outside of the water’s enclosure and drowning it out. Reality came to represent a distant construct for which engagement seemed impossible. It is a sensation of existing within oneself, in a reality that is abnormal, terrifying, lonely, and crushingly hopeless, so much so that resurfacing from this reality genuinely seems impossible—an inconceivable eventuality—and continuing to exist in this state seems equally impossible and undesirable. This is depression’s catch-22.

After more time, weeks and months, activity requiring any effort became increasingly impossible. This soon extended to simple activities like walking, talking, engaging with others, and eventually simply thinking. While at work, at that time I was working in a music shop, I stopped engaging with customers. The sheer effort of having to listen and respond, to a query as simple as the location of a product, or to smile, greet, and acknowledge customers, even for a few seconds, saw me become exhausted, mute, and withdrawn within the first hour of the day. Finally, I simply came home “sick” from work in the mornings, after an hour or so at work, returning to my flat, getting back into bed, and trying to sleep through the rest of the day and night with the blinds drawn and the flat empty and dark—hoping to feel better and “try again” at work the next day. Alcohol factored more in my life at this point, providing me a temporary reprieve from inerratic stagnation brought on by anxiety, dampening my constantly bubbling worries about my declining mood, and allowing me to deny reality for a time. However, ultimately alcohol worsened everything beyond measure. Alcohol would continue to play—with varying degrees of influence—this role in my life until I reached the age of 28, a decade later. Coincidently, on the day I write this paragraph, I have been completely sober from any-and-all alcohol for exactly eleven and a half years to this day.

After several weeks of the above pattern, my parents, somewhat shocked by my gaunt appearance, absent communications, and disinterest, convinced me to attend an emergency doctor’s appointment—my mother accompanying me. It was at this appointment (I had reluctantly attended several prior, always insisting I was “well” but struggling with “tiredness”) that I was diagnosed with MDD, prescribed medication, and signed off work. Notably, then, the stigma of MH in the workplace was at such apex (in Scotland in 2002—at least where I worked and lived) that it was felt it would be “less embarrassing” for me to have “exhaustion” on a medical sign-off certificate. This was initially provided, the certificate switching to “clinical depression” only during the second month of my absence from work.

I was first signed off for 2 weeks, but this became months. The days blended together, me mostly sleeping and remaining in bed. In the first month, I did nothing. I had no contact with any of my friends but one, who once visited me to see how I was doing (I’m still in touch with her, and she is one of the kindest and most empathic humans I’ve had the pleasure to know). I felt the effort required to return a text or voice message represented an impossible, gargantuan feat that I would never be able to conjure—at the time, it was. My parents visited me, bringing me food and taking me to their house regularly, where similarly, I lay in a different bed, but ate more regularly. Usually, these visits ended in me becoming completely withdrawn, going silent and begging to be returned to my flat where the pressures of dialogue were absent, but also, where there was no positive stimulus or company: a double-edged sword.

At this time, I was under the care of weekly doctor visits. After the first month, it was decided that the medication I was taking, at already a high dose, was not working sufficiently. I had already experienced one medication switch early-on in treatment. Now, my current medication would be titrated, increased upward in small incremental doses. This was until I felt I could engage in simple activities: sleep, get out of bed regularly, brush my teeth, and shower. Dosage would be reviewed weekly and side effects evaluated. While I noticed significant side effects, they were of such little care or consequence to me in my present condition that I hardly felt them worth mentioning.

By the second month, nothing had changed. But that’s not true: some of my darker and all-encompassing thoughts had taken on a different quality, and they seemed further back in my mind, less immediate. The dark, crushing, underwater world within which I existed felt the same, but it seemed like my orientation in this world had shifted. I had previously been facing toward the bottom of the diving pool, descending lower into a seemingly endless pressured-chasm of blackness—and only one outcome appeared certain: I would be crushed. Now, I had the sensation that this descent had paused: I had either reached the bottom of the chasm or I had stopped descending. Instead of facing the endless bottom, I was now looking up from the murky depths toward a distant, obscured surface far above. However, I was still sure that I would eventually drown.

At a doctor’s meeting around this time, it was decided that the dose of medication I had reached was high enough that it could not be increased further; the current dose already being evaluated (and approved) on a weekly risk–reward basis for safety. I was to remain on this dose and medication, evaluated every week. Progress would be monitored and a decision about next steps would be made. At this point, there were some discussions of hospital admission, adjunct treatments, and the need for me to begin eating again, but ultimately, I was allowed to go home.

In the weeks that followed, I reached the middle of the third month on high-dose medication. Nothing had dramatically changed. But I began to try and force myself to get out of bed and engage in small activities: having a shower and brushing my teeth, eating two very small meals, and doing five or so push-ups during the course of each day, attempting to build a small routine, and going back to bed after each activity. As I spent most of my time *trying to sleep*, as opposed to *sleeping*, I tried to stop remaining in bed throughout the day. The medication I was taking—at the dose I was taking—did nothing to help my sleep. It increased my insomnia to new levels, only sleeping for a few hours in each 24-hour period. Before I had felt too unwell to feel bored, but now with the kick of artificial energy the medication provided and the months I had spent doing nothing, I had begun to feel under-stimulated.

### DeusEx

It was in this phase of illness that I remembered the PC I had in my flat; the first time in months I had switched it on. I recall brushing the dust from the monitor’s screen, attracted by the discharged static, and wiping the dust from the keyboard. It was a good PC, with a good graphics card. My mother had bought a new PC and had gifted me her old one, but had the kindness to first seek the help of a colleague, who she had paid to upgrade the PC first, so that I wasn’t simply being gifted an outdated computer of little modern gaming use (which I would have still been happy with).

I remember looking through the small collection of games that I owned. While I liked video-gaming, I had not played anything for a long time and had grown to prefer consoles over PC gaming, as was (arguably) the trend of the time. I remembered that I had purchased a game months before: *DeusEx*, on impulse, knowing nothing about the game. I had wanted to purchase *Half-Life*, but at the last minute, I had changed my mind. It was late at night when I installed and first played *DeusEx*. I don’t know why I’m so sure, but I have a firm memory of this, and I recall wanting to play it at a time where it was quiet in the flats around me. I wore headphones when using the PC, plugged directly into the PC-speakers. I recollect not wanting to take them off. It was more comfortable to me to play at night: less anxious, more quiet, still, and controlled. Maybe the headphones reminded me of swimming, being underwater.

*DeusEx* depicts a first-person cyberpunk adventure with role-playing elements, set in 2052: a disturbing, dystopian future landscape; a world arising from societal collapse where threats are ever-present and pockets of safety within this reality are distanced and near-absent. The player takes control of *JC Denton*, an artificially augmented agent for an anti-terrorist unit, with multiple enhancements made due to nanotechnology, the character existing in a world where physical technological augmentations are commonplace, and evolutions are on the cusp of rolling-out new nontechnology augmentations as standard to military and government personnel.

*DeusEx* was immediately enthralling to me, and I recall playing it that first night for hours, until the light began to filter through the blinds and I realized I had lost track of time. This was a turning point for me, a realization. That I had lost track of time meant that I hadn’t been focusing on how I felt. Normally, time seemed to inch by, to almost pause and become stagnant, representing an interconnection with my mental state. I recall feeling different for the first time in months, not specifically from playing *DeusEx* but at the possibility of escapism; I had *lost track* of time—but as I considered time and feelings as nominally connected, I had also lost track of how I felt. *I had escaped*. This possibility excited me for the first time in nearly 4 months. Over the next few months, I played *DeusEx* frequently, always late at night wearing headphones, and I often played until the early morning.

The world of *DeusEx* is complex to explain to those who have not experienced it, and may seem easily outdated, the graphics laughably poor, and the controls “clunky” from the perspective of a modern gamer. However, in 2002, the game provided several unique properties. *DeusEx* felt like playing a film or an episode of *The X Files*. The player controls the protagonist around known cities, depicted in a post-collapse context, for example, the island of Hengsha off the Shanghai coast, Hong Kong, New York City, and London. The conspiracy-driven storyline flits between missions and objectives across these locations, providing immense variety to the gameplay and virtual environments. The storyline of *DeusEx* is enthralling to follow, the player taking on a detective-like role as a double agent and central investigator of a convoluted conspiracy theory. As the game progresses, keeping track of character dialogue, character-affiliations with different government and non-government groups, and stratified objectives, character and group motivations, and requests, all require mental effort, effort I found for the first time in months that I could conjure. Further, the interactive dialogue process for engaging with the multitude of characters in *DeusEx* is masterful. The player selects dialogue from several choices, options continually evolving as conversations continue. All choices have certain conversational consequences, unfolding clues regarding the overarching conspiracy storyline, characters, and goals, but also revealing many side-quests and objectives. Similarly, some choices have negative consequences, causing different characters and factions to shun *JC*, closing off certain storylines and quests. The same principle is applied to how missions and objectives can be completed, with many ways of obtaining information and goals possible, the opposite of the “one-correct-route” formula many games of the time adopted.

A further point, which was near-mind-blowing to me at the time, is that there are a range of different digital book-readers (data-cubes), books, newspapers, computer terminals, and similar objects scattered throughout the *DeusEx* world, allowing the player to read whole sections of dialogue and texts surrounding events within the virtual space, and pertaining in relevance to the storyline and events. Some of these digital objects are real-world extracts from classic books, free from copyright. Thus, the world of *DeusEx* was immersive, and I quickly found myself lost in this world, traversing between the different in-game locations and becoming invested, enchanted, and beguiled by the plot and the twists and turns of the storyline and the characters, settings, predicaments, and occurrences.

Reflecting—within an AE lens—upon my gaming experience, the primary positive effect I experienced was escapism: a vicarious living through the actions of a digital character in a virtual-world of high stimulation and dynamic change that required constant focus. This regularly took me—with each play— away from my real-world mental state. But, the effect was *more*. I felt a distinct synergy between *DeusEx* and myself—the designs and presentations of *DeusEx* seemingly fitting me *perfectly* as an escapist and immersive distraction. Beyond gameplay observations, the design and the color pallet of the game environments closely reflected my mental state. The context of a post-collapse reality was depicted in dark colors, night-time locales, and poorly lit locations; fostering confusion, obscuration, and uncertainty, all emotions I felt. Months earlier, I had attempted to play some console games; brightly lit, hyper-colored, and cartoon-like depictions and worlds, events, and characters that seemed utterly discordant with my state of mind: clashing constructs for which I could find only a complete absence of engagement. *DeusEx* was polar-opposite to this, seeming more like a complete virtual-world to inhabit. Thus, it was the perfect game for me to play and seek escapism within, while still containing peripheral elements that paradoxically anchored me to—and paralleled—my reality, avoiding total discordance. *DeusEx* constructed a liminal, safe, virtual escapist platform for me. This virtual space was not so jarringly different or “fake fun” from my own internal space that it felt impossible to interact with, as other games did, but also not in itself so wildly depressing and “dark” as to promote or amplify existing depressive feelings I felt.

As I entered my fourth month “doing nothing,”^4^ I had been playing *DeusEx* for around a month, several hours each evening. I should qualify “playing”: I would often spend time simply existing within the game-world, wandering, conversing with characters, and reading in-game materials, at times, advancing small side-quests but mostly enjoying the distractions of the virtual-world. Contrasting to the months before, time went by quickly. I found myself renewed with a small inner strength, something I did not expect to experience. I began to complete small real-world tasks, caring for myself, and now also my flat, seeming to leave me now with remaining energy. I began occasionally venturing for short walks, eventually extending these to shopping trips for food, and then short swim sessions. All this time, I maintained my nightly routine of playing a few hours of *DeusEx*, beginning to focus more on advancing the main-quest, but continuing to linger in the virtual-world. I was invigorated by the puzzles and environments, spending much time re-loading save-sessions and re-attempting puzzles and conversations in different ways, marvelling at the different possibilities as I continued the main-quest, now with high-enthusiasm and engagement.

The weekly doctor’s meeting continued, until one week where I felt myself saying that I was feeling a little better. Toward the end of the fourth month, I applied for a new job, and was subsequently offered and accepted this, returning to full-time work toward the beginning of my fifth month after having been signed off from my previous job. In the 6 months that followed, I gradually began to “recover” (enter temporary remission), although I should make clear I remained on a high dose of my primary antidepressant for several years before several years absent from medications.

## Discussion: Reflexive Societal, Identity and Masculinities Reflections on Mental Health, Generated via Feminist-Autoethnography

Historic attachments of gaming as a mostly “masculine” pastime have permeated past societal stereotyping (Bootes, 2024; Newman & Vanderhoef, 2023). This was the case in Scotland in 2002, with gaming viewed as a “masculine” activity linked to notions of being “uncool” or having few friends. This renders the gendered stereotype of gaming-masculinity as a subordinated masculine culture, actively inverse to notions of contemporary HM. Paradoxically, however, many video games of the time exhibit congruence with HM constructs as central archetypes of their design. For example, characters such as *Duke Nukem*, heavily muscled, hypersexual, dominant, violent, stoic, and confrontational, embody a blend of hypermasculine *hyper-hegemonic* characteristics resplendent of hegemonic-masculinity-on-steroids, at times operating to subordinate women, and men performing identities misaligned with hypermasculine depictions. Similarly, HM trends, particularly notions of utilizing narrowly defined “feminizations” as modes of masculine delegitimization exist concurrent to hypermasculine stereotypes.

Games such as *Metal Gear Solid* (MGS) stratify male characters, such as protagonist “Snake” via selective displays of HM, presenting this character archetype as embodying strong physicality coupled with high emotional stoicism, independence, aloofness, and self-reliance among other notions suggested as archetypal of HM, yet—at times—absent of the subordination components required to qualify this descriptor (Connell, 2020; Lucy, 2024). This is contrasted with allied MGS characters such as the scientist “Otacon,” positioned as physically slight and readily emotional; openly struggling with emotive themes and occurrences, Snake remaining resolutely stoic by comparison. While there is no direct subordination, the polarization of masculine motifs is utilized to construct divorcing effects. HM as influencing gendered virtual depictions can also be uncovered when considering patriarchal processes: the *male gaze* perspective linked to some video game depictions of female characters from the 2000s. This thinking is well-established (Hoffswell, 2011). Notable examples from my own memory include (the obvious) depictions of Lara Croft in the *Tomb Raider* franchise, existing as an archetypally “male” depiction of a “strong female lead,” representing (arguably) idealized male-sexualized physicality and presentation, but balancing this with independence, emotional, and physical strength and intelligence. Others have discussed this dichotomy (Mikula, 2004). Similar male-gaze-led depictions, that also contain the above dichotomies, are evident in depictions of *Jill Valentine* and *Claire Redfield*—both leading protagonists from the *Resident Evil* franchise, displaying (again arguably) male-idealized physicality and presentations, but embodying notions of strength, resilience, intelligence, and skill. While depictions resist gendered-typing when applying a contemporary lens; society comprising many masculinities and femininities—all legitimate and performing identities and acts in manifold intersecting ways and embodiments, historic depictions of stratified gender-typing are found in gaming contexts that separate and simplify characters based on proximity and distance from HM-like archetypes.

Contrasting with gendered reductionisms, I found *DeusEx* different. Characters were rarely singular-dimensional, composed of complex back-stories with multiple threads of personality. While emotional stoicism factored heavily as a plot-point for *JC*—the *DeusEx* protagonist—the variety of player-choice options allowed for players to enact their own identities within the game-universe, resisting stoicism in a range of different ways via diverse in-game choices and dialogue decisions. Thus, playing *DeusEx* represented less of a discordant experience with my mental state at the time. Unpacking this, as I have discussed above, the primary positive effect I experienced from *DeusEx* was one of escapism; however, this can be readily broken when one is forced, via gameplay mechanics, to engage in choices and activities that are discordant with their own thinking, beliefs, and values, lending to a rejection of the gaming medium as a positive, escapist tool.

I now apply this theorizing to self-examining my feelings regarding motifs of high-stoicism, exploring why these construct identity discordance.

### Autoethnographically Examining Masculine Stoicism

When I was acutely unwell, in 2002, male MI was seen as something to be ashamed of. Reflecting, this was heavily routed in patriarchal notions of viewing MI, and even admittance and discussion of MI, as “weak” or “less masculine.” This operated in congruence with Connell’s notions that men performing practices counter-to-HM attract attachments of marginalization and subordination, these behaviors being typed as “feminine,” inverse to archetypal masculinity (Connell, 2020).

For my own MI experience— to be clear, excluding my very supportive immediate family—most attempted discussions regarding MI I had with male friendship circles, employers, and acquaintances led to a devaluing of any “masculine” legitimacy or “social currency” I may have been perceived to carry (by others). I was told people “do not want to talk” about men experiencing MI. Part of this may have been due to attitudes surrounding MI, myself and my peers being children of the early mid-80s, and thus brought up in an era and location which arguably had little overall recognition—at all—for MH. Most discussions of MI I attempted saw my dialogues attributed to engaging in displays of “over-emotional,” “sensitive,” and “strange” subject matter that depicted and confirmed a failure of the supposed male stoic character: the slipping of a mask that should be upheld, as opposed to a practice of required help-seeking and search for confirmation, legitimization, recognition, empathy, and solidarity from others (other men). Some people simply went silent, changed the subject, or reacted with disbelief when I attempted to discuss MI with them. Others attributed diverse causal factors to my MDD, suggesting this was caused by interest in spending too much time alone as opposed to time alone representing a side effect of MDD.

The silence of others (other men) and the “shutting out” of MI discussions was also maintained in other—more insidious—ways. When trying to speak to some of the men closest to me, processes of silencing, conversational diversion/re-routing, and comparisons were invoked to immediately truncate, halt, and prevent discussion. Upon trying to discuss my MDD with one man, I raised the importance of men speaking openly. This was met with the firm comment: “Well. You certainly speak about it.” This immediately ended the discussion, me feeling chastised for trying to broach the taboo topic of support and help. Speaking with another male individual, I recall another attempt at opening-up. This was met with: “OK, but to change the subject …”—the subject then shifting to a different “happy” conversational theme, as if what I had said had not been heard at all. This reinforced notions that discussing MI and MDD was “strange” and inappropriate; the immediate—forced—shift in conversation and ensuing awkwardness demonstrating to me my extreme social faux pas. I recall other times where I raised the issue of MDD and was presented immediately with reductive comparison scenarios: “There’s nothing to be depressed about, you’re strong: fit and healthy.” This extended to overt ableist scenarios where my “mental issues” were compared to the physical disabilities of others: “It’s all in the mind. I saw a guy […] who had both legs amputated, and he was still able to be happy.” While this statement is offensive both for its ableist and identity reduction connotations, I utilize this example to illustrate some prevailing thinking regarding MI as perceived “not as severe” as physical disabilities in the eyes of some (see McRuer, 2006).

The above occurrences reinforced a lifetime of prior conditioning, assimilation, and accommodation of MI opinions, media portrayals, and societal norms regarding MI—for men—being portrayed as a weakness of masculine character and a malfunction in acceptable performances of male identity. This detracts from archetypal HM-linked notions of stoicism, emotional regulation, and control, and due to the debilitating effects of depression, also a loss of physical prowess, ability, and bodily maintenance and strength. Thus, to be depressed (within this time and context) constituted a forced performance of heterodoxy against the dominant sphere of HM. To clarify, to be depressed was to cease being a man in any acceptable or recognizable form for this temporal–social context and societal bubble. While one may not have been performing HM, to be depressed cancelled-out ones’ *pursuit* of HM--praxis Connell (2020) argues *most men* engage in to be socially accepted, bond and avoid marginalization. Like Drummond’s (2010) exploration of masculinity and physicality in sport, masculine stoicism is double-edged, promoting societally desirable (non)performances of masculinity on the one hand, preserving HM-like-reverence, but degrading and decaying many other aspects of performance required to uphold this HM-like reverence: physicality, control, and independence. As such, in my teens and early 20s, I believed that to “admit” to struggling with MI represented at its core a failure in all socially performative norms that depicted acceptable masculine character and thus represented my complete failure as a human being. Likewise and again based on this conditioning, I resisted help-seeking for a long time, self-medicating and perceiving my own MDD struggles as “a choice” of my own making; something to be deeply ashamed of and kept secret. This thinking was amplified and reinforced in numerous interpersonal and institutional ways in the early 2000s, from being advised “not to tell anyone” about my MI and medication usage to disclosures of my MDD experiences leading to social rejections.

Over time and with much help, I have somewhat successfully reversed internalized thinking of MI as “my fault.” However, it is important to acknowledge that “undoing” lengthy, early-life, longitudinal social conditioning is effortful. This has largely been made possible only by the dilution and devaluing of HM itself as a structuring social principle within the wider societies and temporalities within which I am situated, masculine norms now being wildly different. Importantly, the key points from the above are less the challenges of my own experience. Despite the AE focus of this work, I remain unimportant. Speaking to Fisher’s comments of sharing his own experiences of depression:

I offer up my own experiences of mental distress not because I think there’s anything special or unique about them, but in support of the claim that many forms of depression are best understood—and best combatted—through frames that are impersonal and political rather than individual and “psychological.” (Fisher, 2014a, p. 1)

In this same spirit, my central contribution must be considered for the spotlight it shines on the male MI perspective. While revisiting shared narratives has been emotionally challenging, I have done so primarily to de-stigmatize this sharing experience for others. I put forward that many men have experienced early conditioning, internalization of stigma, and programmed resistance toward help-seeking for MI. While society often claims to have moved beyond propagating such stigma for men, prior masculine conditioning embodies a unique *hauntological* quality (Fisher, 2012, 2014b). That is, the specter of historical conditioning and its structural effects still lingers and exerts significant influence over present thinking, male self-talk, acceptance, and (in)actions. I fear the reality of my experiences as a young man trying to discuss MI with other men 22 years ago is still very much depictive of some spheres of contemporary reality for many men today. While this is an immediately uncomfortable position to admit in the present temporality, I would argue—as a man who still experiences MI as MDD and as a research psychologist—that the oft-propagated media and societal fantasy of *most* men now immediately being comfortable speaking-up and seeking-help when experiencing MI is distant from the lived experiences of many men.

To this point, the *hauntological*, spectral, and echoing effects of stoic masculine norms imprinting upon men must be considered **real**. It is unhelpful to suggest—as some have publicly done—that men need to “let go” of imprinted stoic notions surrounding MH and adapt to a shifting reality; readily, openly, and immediately now discussing their mental concerns without anxiety, stigma, issue, or sense of internal identity contradictions. While this is an ideal and desirable position, it is not realistic. If unlearning such ingrained and semi-permeable longitudinal conditioning could be achieved with a click of the fingers, most men would readily engage this unlearning. A more useful positional analysis may be achieved by considering the much-more-readily accepted position that ingrained societal patriarchal processes still exist and disadvantage, marginalize, and subordinate other genders. These must be structurally reconfigured as a societal imperative. Yet, it is lacking that the permanence and enduring qualities of these structures are readily accepted in non-male disadvantaging contexts, while the effects of such axiomatic, taken-for-granted, and patriarchal structures upholding (as Connell, 2020 argues) *most men’s positioning toward revering* HM that reflect to constrain men’s MH help-seeking are—at times—downplayed and questioned. It is critical for all humans that HM processes are dismantled in society, and as a component of this, men **reconfigure and unlearn** notions congruent, supporting and legitimizing HM and HM-like thinking that generates resistance toward help-seeking for MI. However, this position will not be achieved by downplaying the difficulties of unlearning a lifetime of social conditioning longitudinally upheld as normative by patriarchal society.

## Conclusion

In conclusion, my own experiences with video-gaming through MDD generate several learning perspectives. Firstly, the feminist-autoethnographic lens employed for self-examination has allowed me to speak candidly about my own experiences, detailing how *DeusEx* offered an escapist point of salvation for me; a window of distraction, while medication took effect to lift me from my MDD state. On reflection, I wonder if I had not had this distraction, whether the medication would have worked, such was the feeling of relief upon realizing I could temporality escape the mental space within which I had found myself. Reflecting on MI, MDD, and anxiety, and in keeping with the honest and open principles of AE, I still experience and struggle with MI, and my journey regarding this has been complex and lengthy and has involved several diagnoses and numerous medications, also some periods managing without medications, and trailing medication combinations; graduating in the last decade well beyond the realms of traditionally prescribed medicine to more “niche” and specialist antidepressant options: finally, MAOIs.^5^ However, among coping mechanisms, video-gaming has remained a formidable tool for fostering escapism. Scholars may utilize my AE perspectives on video-gaming through MDD as a means of widening conversation regarding possible therapeutic effects of video-gaming, particularly escapism, temporal relief, and distraction. Perspectives are under-researched and under-discussed yet represent a valid avenue of investigation deserving more scholarly enquiry. Reflecting on theoretical implications, this manuscript raises salient structural issues regarding notions of HM and the *hauntological* qualities of social conditioning that mold men’s resistance surrounding acknowledging, speaking-up, and help-seeking vis-à-vis MH. While society—at times—propagates suggestions of ubiquitous openness, the echoes of previously enforced conditioning must be recognized as constructing ongoing restrictions. It is my hope in using AE, and recognizing that there are nearly no discoverable AE MH perspectives published by men, that this paper promotes further use of the AE method for sharing, and self-and-collective examination of men’s MH experiences and the unique issues faced by men with regard to progressing MH acceptance and developing wider scholarly discussions.

## Notes

1. I use the term “mental illness” to describe my journey with long-term depression and anxiety. I recognize this term may be ill-fitting to describe these conditions in all cases; however, it applies in the localized case I refer to for most of this work; my MI experience rendering me more unwell than any physical illnesses I have experienced.

2. I have placed “recovery” in double inverted commas, highlighting ambiguity surrounding the term. It would be more accurate, per my own lived experiences, to describe my MDD recovery as a transient process of temporary remission, having been treated for MDD and associated illness intermittently but recurrently for over 22 years.

3. I should be clear about my diagnoses: I have been diagnosed formally with ADHD in childhood and again formally as an adult. I was diagnosed with dyspraxia as a child. In addition to past diagnoses of MDD, treatment-resistant and atypical depression, I have also been diagnosed in the past with anxiety and obsessive tendencies (particularly around routine, ordering activities, food, and exercise). I am presently progressing through the formal diagnostic pathway for autism. It has previously been suggested (as a child and adult at various diagnostic meetings) that I was/am high-functioning anxious and/or high-functioning autistic. While I largely resist static diagnostic labels in favor of adopting intersectional identity perspectives to make sense of myself, I feel I should be transparent and disclose these diagnoses to highlight the complexities of my (and thus others’) MH experiences as manifold rather than use the singular descriptor of “depression” or “major-depression” to encompass a complex and varying set of experiences.

4. I have decided to use double inverted commas around “doing nothing.” This is to highlight the ambiguity of the term for those experiencing depression and recognize that some may be frustrated at the use of this term without context. While I have mentioned “doing nothing” several times previously, highlighting the lack of structure to my days during this time, my days were not spent “doing nothing” in the traditional sense. They were spent surviving, thinking, resting, processing and attempting to “recover,” and dealing with failed attempts at this—all of which carried a significant effortful mental burden. Thus, resource for physical activity during this period was near-absent.

5. MAOI stands for monoamine oxidase inhibitor. MAOIs are an older, separate class of medications from other antidepressants. MAOIs have dietary restrictions and unique side effects and are primarily used for treatment-resistant depression when other medications have little or no therapeutic effect.
